# Error Processing in Huntington's Disease

**DOI:** 10.1371/journal.pone.0000086

**Published:** 2006-12-20

**Authors:** Christian Beste, Carsten Saft, Jürgen Andrich, Ralf Gold, Michael Falkenstein

**Affiliations:** 1 Leibniz Research Centre for Working Environment and Human Factors, WHO Collaborating Centre for Occupational Health and Human Factors Dortmund, Germany; 2 Department of Neurology, Huntington Centre NRW, St. Josef Hospital, Ruhr-University Bochum Bochum, Germany; James Cook University, Australia

## Abstract

**Background:**

Huntington's disease (HD) is a genetic disorder expressed by a degeneration of the basal ganglia inter alia accompanied with dopaminergic alterations. These dopaminergic alterations are related to genetic factors i.e., CAG-repeat expansion. The error (related) negativity (Ne/ERN), a cognitive event-related potential related to performance monitoring, is generated in the anterior cingulate cortex (ACC) and supposed to depend on the dopaminergic system. The Ne is reduced in Parkinson's Disease (PD). Due to a dopaminergic deficit in HD, a reduction of the Ne is also likely. Furthermore it is assumed that movement dysfunction emerges as a consequence of dysfunctional error-feedback processing. Since dopaminergic alterations are related to the CAG-repeat, a Ne reduction may furthermore also be related to the genetic disease load.

**Methodology/Principle Findings:**

We assessed the error negativity (Ne) in a speeded reaction task under consideration of the underlying genetic abnormalities. HD patients showed a specific reduction in the Ne, which suggests impaired error processing in these patients. Furthermore, the Ne was closely related to CAG-repeat expansion.

**Conclusions/Significance:**

The reduction of the Ne is likely to be an effect of the dopaminergic pathology. The result resembles findings in Parkinson's Disease. As such the Ne might be a measure for the integrity of striatal dopaminergic output function. The relation to the CAG-repeat expansion indicates that the Ne could serve as a gene-associated “cognitive” biomarker in HD.

## Introduction

Huntington's disease (HD) is an autosomal dominant, monogentic neurological disorder causing a degeneration of the neostriatum. The disease is genetically expressed by an extension of the CAG-repeat length at the 4^th^ chromosome [1] encoding a large protein, the “huntingtin”. This protein accumulates and causes apoptotic striatal neuronal death [2]. The most obvious sign of HD is chorea: rapid, arrhythmic and complex involuntary movements, which are supposed to be an effect of dysfunctional error-feedback processing [3]. Besides these motor symptoms, psychiatric and cognitive deterioration appear, finally associated with dementia [4]. HD is accompanied by alterations in the dopaminergic system with a reduction in D1 and D2 receptor density [5], [6]. It is shown that the decreased striatal dopamine receptor content is related to CAG-repeat length [7], [8]. Also animal studies show a close relation of CAG repeat expansion and dopaminergic alterations [9]. Yet besides dopaminergic alterations, other neurotransmitter systems are also affected [10] and neuroanatomical degeneration is wide spread [11].

The dopaminergic system itself is involved in many cognitive processes. One such process most likely depending on the dopamine system is the processing of errors and hence performance monitoring. A means to assess error-related processes is via an event-related potential (ERP), called error negativity (Ne) [12] or error-related negativity (ERN) [13]. A major source of the Ne is located in the medial frontal cortex, especially the anterior cingulate cortex (ACC) [14]–[16]. The Ne is classically interpreted as the detection of a mismatch or conflict between response representations [12], [13], [16], [17]. Another theory [18] assumes a more general functional significance of the Ne. According to this “*reinforcement learning hypothesis*” the midbrain dopamine system (DA-system) supervises and evaluates evolving events, such as responses. If an event is not as expected (e.g. an incorrect response), the DA-system sends an error signal to the ACC which in turn elicits the Ne. There is converging evidence that the Ne is in fact dependent on the dopaminergic system [16], [18]–[22]. In particular, the Ne is reduced in the most common basal ganglia disease, Parkinson's disease (PD) [22], but also a null result is reported [23]. Further, the Ne was found to be reduced in patients with focal basal ganglia lesions [24].

The goal of the present study is to investigate whether patients with HD also show alterations of the Ne as shown in PD. Although, the fundamental deficit is different in the two diseases, i.e. in PD the primary deficit is a reduction of DA-producing cells in the substantia nigra, hence leading to a presynaptic deficit. In HD striatal cells are affected, hence leading to a (post)synaptic deficit. Finally, both Parkinson's and Huntington's disease compromise striatal output function. If the Ne depends on an output signal from the basal ganglia to the ACC, this should also cause a reduction of the Ne in HD. Such a finding would strengthen the evidence that the Ne is related to the integrity of the dopaminergic striatal output and may serve as a measure of this integrity, maybe even define a gene-associated biomarker in HD, since dopaminergic alterations in HD are related to genetic factors (e.g. CAG repeat expansion) [7]–[9].

## Methods

### Participants

Eleven right-handed, unmedicated HD-patients (N = 11) from 26 to 57 years of age [mean = 39.81, SD = ±8.96] genetically confirmed and with manifest symptoms [25] participated in the study. A clinical description of the HD-patients is given in Table 1.

**Table 1:**
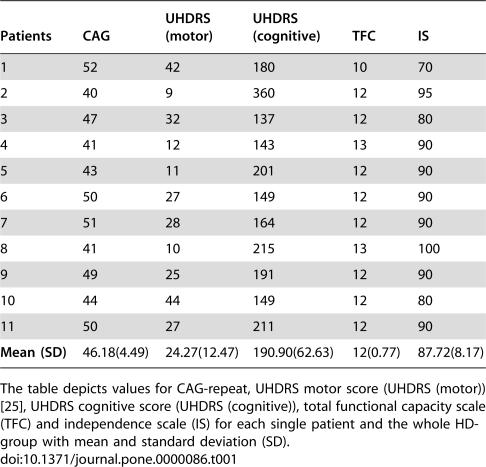
Clinical description of the HD-patients.

Patients	CAG	UHDRS (motor)	UHDRS (cognitive)	TFC	IS
1	52	42	180	10	70
2	40	9	360	12	95
3	47	32	137	12	80
4	41	12	143	13	90
5	43	11	201	12	90
6	50	27	149	12	90
7	51	28	164	12	90
8	41	10	215	13	100
9	49	25	191	12	90
10	44	44	149	12	80
11	50	27	211	12	90
**Mean (SD)**	46.18(4.49)	24.27(12.47)	190.90(62.63)	12(0.77)	87.72(8.17)

The table depicts values for CAG-repeat, UHDRS motor score (UHDRS (motor)) [25], UHDRS cognitive score (UHDRS (cognitive)), total functional capacity scale (TFC) and independence scale (IS) for each single patient and the whole HD-group with mean and standard deviation (SD).

Psychiatric assessment showed that there was no manifest depression as indicated by the *Beck Depression Inventory* (BDI) [mean = 6.54; SD = ±4.36]. Assessment using the *Young Mania Rating Scale* for adults (YMRS) revealed no mania [mean = 4.63; SD = ±3.50]. Cognitive screening for dementia using the *Mini Mental Status Examination* (MMSE) revealed no dementia [mean = 27.09; SD = ±2.11]. Furthermore 12 healthy controls were from 26 to 57 years of age were recruited (mean = 38.12; SD = ±7.56). The same psychiatric assessment using the BDI revealed no depression [mean = 1.83; SD = ±1.46] or any mania [mean = 1.16; SD = ±0.83] as revealed by the YMRS. Both groups had a comparable educational background. All participants gave written informed consent. The study was approved by the ethics committee of the University of Bochum.

### Task

To measure error-related processing we used a “*Flanker Task*” [26] which reliably yields a high percentage of errors. This task was similar to one of the three tasks used in the cited PD-study [22]. In the present task vertically arranged visual stimuli were presented. The target-stimulus (arrowhead or circle) was presented in the center with the arrowhead pointing to the right or left. The central stimuli were flanked by two vertically adjacent arrowheads which pointed in the same (compatible) or opposite (incompatible) direction as the target. The flankers preceded the target by 100 ms to maximize premature responding to the flankers, which would result in errors in the incompatible and Nogo condition. The target was displayed for 300 ms. The response-stimulus interval was 1600 ms. Flankers and target were switched off simultaneously. Time pressure was administered by asking the subjects to respond within 550 ms. In trials with reaction times exceeding this deadline a feedback stimulus (1000 Hz, 60 dB SPL) was given 1200 ms after the response; this stimulus had to be avoided by the subjects. Four blocks of 105 stimuli each were presented in this task. Compatible (60%) and incompatible stimuli (20%) and Nogo-stimuli (circle) (20%) were presented randomly. The subjects had to react with the thumb depending on the direction of the central arrowhead and to refrain from responding to circles. The Nogo trial data were not further evaluated within the present study, which focused on error processing and not on inhibition.

### EEG recording and analysis

During the task the EEG was recorded from 32 electrodes (Ag/AgCl) (Fpz, Fp1, Fp2, Fz, F3, F4, F7, F8, FCz, FC3, FC4, FC5, FC6, Cz, C3, C4, C7, C8, Pz, P3, P4, P7, P8, Oz, O1, O2, M1, M2), two lateral and four vertical EOG electrodes (sampling rate: 500 Hz). Cz was used as primary reference. The filter bandwidth was from DC to 80 Hz. Impedances were kept below 8 kΩ. The EEG was digitally filtered using a 0.10 Hz high-pass and 20 Hz low-pass filter. From the EEG response-locked ERPs were computed, beginning 400 ms before and ending 700 ms after the correct or incorrect response. After this, eye movement artifacts were corrected with the Gratton-Coles-Algorithm using the EOG data [27], followed by a baseline correction (−200 ms - 0 ms [i.e. response]). Remaining artifacts were rejected using an amplitude criterion of ±80 µV followed by re-referencing all data to linked mastoids. The Nogo trial data were not further evaluated. The amplitude of the Ne in error trials and of the CRN in the correct trials was measured relative to the peak of the positivity, which precedes both components [26], [28], [29].

### Statistics

Peak-to-peak amplitudes were subjected to a repeated-measures ANOVA with electrode (Fz, Fcz, Cz) and correctness (right/false) as within-subject factors and group (HD, controls) as between-subject factor. The degrees of freedom were adjusted using the Greenhouse-Geisser-Correction when appropriate. In addition, separate univariate ANOVAs of the post-response negativities after false (i.e. the Ne/ERN) were conducted at electrodes Fz, FCz and Cz with the between-subject factor group. For these analyses Bonferroni-corrections were applied. Tests of normal distribution using the Kolmogorov-Smirnov Test revealed that each variable included to the ANOVAs was normal distributed (all z<0.883; *p*>.205; one-tailed). Because of higher test-power, one-tailed tests were used. As a measure of variability the standard deviation (SD) together with the mean is given.

## Results

### Behavioral data

The analysis of the reaction time (RT) revealed that the HD-group reacted more slowly than the control group in correct responses (c-RT) [HD: 420.74 ms±13.96] [controls: 324.19 ms±13.36] (F(1,219 = 24.94; *p*<.001). The same was apparent for the error responses (f-RT) [HD: 330.34 ms±60.66] [controls: 259.12 ms±35.59] (F(1,12) = 12.04; *p* = .002). Both groups did not differ with respect to the frequency of errors [HD: 20.72±9.50] [controls: 25.33±10.25] (F(1,21) = 1.24; *p* = .278). RTs of correct responses after an error was committed (post-RT) are generally prolonged, which reflects the behavioral adaptation after an error. Therefore we subjected the mean reaction time of all correct responses and those after an error as within-subject factor to a repeated measure ANOVA with group as between-subject factor. Post-RTs [402.41 ms±16.27], were significantly longer than c-RTs [372.46 ms±9.66] (F(1,21) = 7.26; *p* = .014); no interaction with the factor group (F(1,21) = 1.37; p = .255) was obtained.

### Electrophysiological data

**Figure 1:**
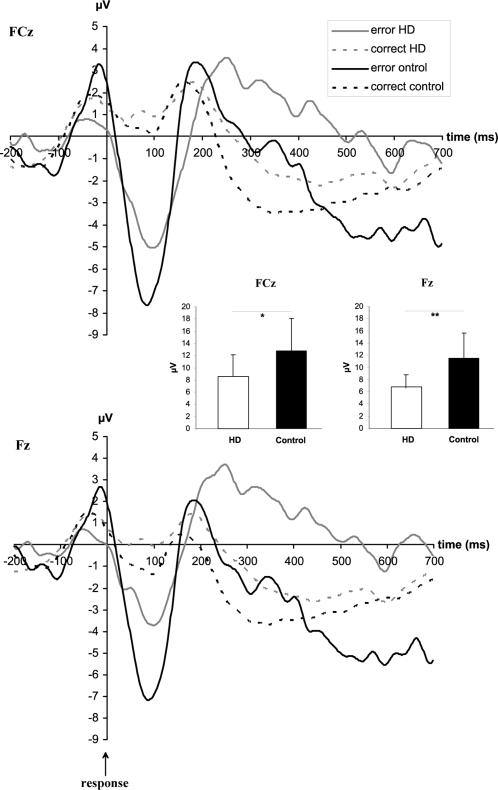
Event-related potentials of the Ne/ERN and CRN. The Ne/ERN (false responses) and CRN (correct responses) for HD and controls at electrode FCz (top) and at Fz (bottom). The x-axis denotes time in milliseconds (ms). The y-axis denotes voltage in µV. The bar plots denote significant differences in the peak-to-peak amplitude of the Ne/ERN between the groups for the electrodes FCz (left) and Fz (right).

The potentials of the Ne and the CRN are given in Figure 1. The response-related negative potential differed significantly between the electrodes (F(2,42) = 41.61; p<.001). Response-related negativities were larger at Fz [−6.49 µV] and FCz [−7.05 µV] compared to Cz [−3.62 µV] (*p*<.001). Fz and FCz did not differ from each other (*p* = .331). Furthermore the groups differed with respect to the activity at the different electrodes, as reflected in a group by electrode interaction (F(2,42) = 5.75; *p* = .006). As expected, the brain potentials strongly differed between correct and error responses (F(1,21) = 37.70; *p*<.001). This effect differed between groups, as reflected in a group by correctness interaction (F(1,21) = 5.56; *p* = .028). A subsequent simple-effects analysis revealed that the factor group was significant for error responses (F(1,21) = 8.39; *p* = .009) but not for correct responses (F(1,21) = 0.009; *p* = .925). The separate ANOVA for the Ne (i.e. error responses) at electrode FCz (where the Ne is usually maximum) showed that the Ne was smaller in HDs [−8.61±3.51] than in controls [−12.75±5.27] (F(1,21) = 4.78; *p* = .040) (Fig. 1 top). Similar but even larger effects were seen at Fz [HD: −6.70±2.20] [controls: −11.56±4.10] (F(1,21) = 12.19; *p* = .002) and Cz [HD: −3.34±0.90] [controls: −5.48±1.80] (F(1,219 = 12.51; *p* = .002) (Fig. 1 bottom). To check the possibility that the reduction of the Ne in HD patients is due to a larger latency variance we measured the Ne latency in single low-pass filtered error trials [22]. The intrasubject variances of the negative peak latencies for the electrode Fz and FCz were subjected into a repeated measures ANOVA with the between-subject factor group. The intrasubject variances of the negative peak latencies did not differ neither between the electrodes (F(1,21) = 0.005; *p* = .943) nor when the factor group was taken into account, too (F(1,21) = 1.008; *p* = .327). The main effect group showed that both groups also did not differ with respect to intrasubject variances (F(1,21) = 2.88; *p* = .104). The most well-known ERP is the P300. Analyzing this component at electrode Pz across the time window from 200 till 500 ms revealed no difference between the groups [controls: 9.22±0.93] [HD: 7.15±0.97] (F(1,21) = 1.60; *p* = .219).

**Figure 2:**
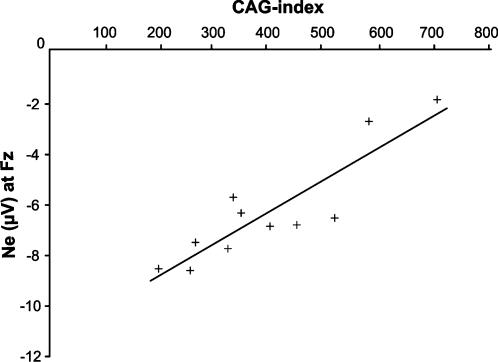
The Relation of the Ne peak-to-peak amplitude at Fz and CAG-index. This figure shows a linear relation of the Ne peak-to-peak amplitude at electrode Fz with the CAG-index, derived via (CAG_n_ – 35.5) × “age of the patient” [see: 2], 30].

In a second step we analyzed the relation of genetic factors to the Ne. For this purpose we used the CAG-index, i.e. the number of triplets in excess (CAG_n_ – 35.5), multiplied by the age of the patient. The CAG-index is an expression of genetic disease load normalized to each individual [2], [30]. The mean CAG-index was 403.81 (SD = 154.34). For correlational purposes we used electrode Fz (which showed the maximum group effect on Ne amplitude) and FCz (the usual maximum of the Ne). Pearson-correlation revealed that the CAG-index was correlated with the peak-to-peak amplitude of the Ne at Fz (*r* = .872, *R* ^2^ = 0.75; *p*<.001) (Figure 2).

A similar but smaller correlation was found for FCz (*r* = .603, *R* ^2^ = 0.36; *p* = .025). However, one can argue that this correlation might be driven by two outliers, but a further analysis excluding these outliers also showed a significant relation of the Ne at Fz (r = .645, *R* ^2^ = 0.40; *p* = .030) and FCz (r = .371, *R* ^2^ = 0.13; *p* = .045). The Ne is known to show age-dependent variations [22], which might be critical when using the age of the patient in the calculation of the CAG-index. However, the repeat itself was correlated to the Ne at Fz (r = .663, *R* ^2^ = 0.43; *p* = .013) and FCz (r = .541, *R* ^2^ = 0.29; *p* = .043) indicating that “age” is not the driving factor in this correlation. This is supported by the fact that even “age” itsself was uncorrelated with Ne at Fz (r = .050, *R* ^2^ = 0.25; p = .442) and FCz (r = −.124, *R* ^2^ = 0.14; *p* = .358). Furthermore it could be argued that the Ne reflects the progression of disease and only to a lesser extend the genetic processes. To meet this objection we calculated the correlation of the Ne with the time span since motor age of onset, as an estimate of disease progression. It can be seen that this factor was not related to the Ne at Fz (r = .379, *R* ^2^ = 0.13; *p* = .125) and FCz (r = −.099, *R* ^2^ = 0.08; *p* = 386).

Besides CAG-repeat length, parental age of onset [31], [32] as well as paternal vs. maternal transmission [31] is of strong influence in and presumably reflects additional genetic and/or environmental influences [33]. Therefore we subjected CAG-index, parental age of onset as well as paternal vs. maternal transmission as independent variables to a stepwise linear regression analysis with the Ne/ERN as dependent variable. The results show that the incorporation of these additional parameters did not increase the amount of explained variance, as shown by the partial correlations (parental age of onset: r = .605; *p* = .112) (paternal vs. maternal transmission: r = −.063; *p* = .882).

## Discussion

In this study we examined an ERP related to error-processing (Ne) likely dependent on the dopaminergic system in HD and its relation to genetic factors. Deficits in this cognitive function are assumed to play a major role in the emergence of motor symptoms in HD [3]. We showed that (i) the Ne was reduced in HD compared to healthy controls and (ii) that this alteration was specific for the error trials (i.e. for the Ne) and related to genetics.

The behavioral data indicated that both groups committed a comparable amount of errors. Thus the group differences in the ERP are unbiased by the frequency of errors. Furthermore the HD group showed no psychiatric pathology (e.g. depression), which might also have influenced the results [34]. The behavioral data of the present study suggest that the HD patients are able to perform behavioral adaptation; in spite they have a reduced Ne. This is in line with the finding that healthy old subjects usually show a larger post-error slowing than young controls, despite a clear reduction of their Ne [22], which either suggests that the process reflected in the Ne is not driving the posterror slowing, or that it operates adequately also in the patients, even when its strength is reduced.

The pattern of results, namely a reduced Ne but unaffected CRN closely resembles findings with Parkinson' disease [22]. In those patients the Ne, but not the CRN (and the Pe [35]) were reduced compared to healthy control subjects. This strongly suggests a similar mechanism underlying the Ne decrease in Parkinson's and Huntington's disease. The reduction in the Ne in HD can be explained by the alterations in the dopaminergic system, which is prominent in HD [5], [6], because the Ne and hence error-processing is determined by the dopaminergic system as stated by other findings in psychiatric and neurological diseases [16], [18]–[22], [36]. The importance of the dopaminergic deficit is underlined by the finding that the P300 did not differ between the groups, which has consistently been shown to be unrelated to the dopaminergic system [37]. As already mentioned in the introduction, the pathophysiology in Parkinson's and Huntington's disease is different: in PD the primary alteration is a depletion of dopamine, i.e. a presynaptic dysfunction. In contrast, in HD, the deficit lies in the striatal cells themselves. It is well documented in HD that underlying genetic factors (i.e. CAG repeat length) are related to dopaminergic alterations at the receptor level [7]–[9], i.e. a strong reduction of DA receptor density in the striatum. Hence we assume that the most likely common cause of the Ne reduction in PD and HD is an impaired functioning of striatal DA system, either via a presynaptic DA depletion (in PD) or a postsynaptic (receptor) deficit (in HD). As such the Ne migth serve as an easy deriveable measure of the integrity of the dopaminergic striatial output function. The fact that the Ne was related to the CAG repeat mutation, which is in turn related to the receptor density/integrity [7]–[9], further supports the dependence of the Ne on striatal DA system (receptor) functioning. This interpretation is supported by older findings in HD in which there was no change in the levels of free-available dopamine [38], [39] that could have influenced the results. Furthermore the findings of an altered Ne in ADHD [19] and the effects of alcohol consumption [18], [21] are also in line with our interpretation, because ADHD is known to show alterations at the receptor level and the effects of alcohol are mediated at this level, too. However, besides the fact that striatal cells are affected in HD, which might lead to the reduction of the Ne, research indicates that also the functioning of the ACC itself is altered [40], [41]. Since the ACC is known to be a source of the Ne, the reduction of the Ne could also be due to dysfunction in the ACC. Although the precise neuroanatomical location underlying the Ne-reduction is uncertain it is likely that the dopaminergic deficit is the driving force for the Ne-alteration in HD. Irrespective of anatomical location (striatum or ACC), the dopamine system (receptor) reduction seems to be of importance.

As stated above we found a correlation of the Ne with the underlying genetic alteration in HD suggesting for a relationship of genes and cognition. Since the CAG-index is age-related, it might be argued that the correlation simply reflect the reduction of the Ne with advanced age [22]. However, even when we used the CAG-repeat (which is independent of age), the correlation remained significant. Also, we found no significant correlation between the age of the subjects and Ne amplitude. The correlation with the Ne amplitude can also not be attributed to be biased due to the progression of disease, since this parameter was not related to the Ne. This strongly suggests that age or disease progression per se cannot explain the high correlation found in our study. As such the relation indicates that the Ne might be used as a gene-associated cognitive biomarker in HD. We assume that this is possible in this case, because a monogenetic factor (CAG-repeat) influences the integrity of the dopaminergic (receptor) system in the basal ganglia. This in turn influences error processing measured by the Ne. The dopaminergic alteration serves as a mediator between genetic and cognitive processes. Generalizing the current results of the genetic relation of the Ne it may be hypothesized that any cognitive process can be related to genetic factors, if it relies on predominantly one neurofunctional system with this system prevailingly being influenced by genetics. However, we cannot completely rule out that changes in other neurotransmitter systems [10] or the wide spread neuropathology [11] also have an influence, but because of the accordance of these results with results in Parkinson's disease and the relation to the genetic factors, we think that the dopaminergic pathology is most important. In similar vein, the limited sample size may also possibly seem critical.

### Perspectives

An important practical consequence from the results is that a simple electrophysiological measure as the Ne might be useful to evaluate dopamine (receptor) functioning in the striatum, or the effects of pharmacological treatment [42], [43]. Since we were not able to include a direct measure of the dopamine receptor integrity (e.g. via PET scans) this may seem somewhat speculative. Hence we think that electrophysiological techniques (e.g. ERPs) can have useful applications in interdisciplinary neuroscientific research. Based upon the current results further research should focus on the presymptomatic stage to disentangle possible *early* “cognitive biomarkers” that might lead to a better understanding of the processes taking place in this phase and might have useful clinical applications in presymptomatic disease care. Furthermore the relation to other biomarkers in HD [44] should be evaluated.

### Conclusions

In the current study we accounted for a reduction of a cognitive event-related potential (i.e. Ne) in HD, which was related to the underlying genetic alteration. Thus the Ne may be treated as an easy derivable gene-associated cognitive biomarker in HD.
